# Antecedents of organizational engagement: exploring vision, mood and perceived organizational support with emotional intelligence as a moderator

**DOI:** 10.3389/fpsyg.2014.01322

**Published:** 2014-11-18

**Authors:** Edward G. Mahon, Scott N. Taylor, Richard E. Boyatzis

**Affiliations:** ^1^Information Technology, Kent State UniversityKent, OH, USA; ^2^Management Division, Babson College, Babson ParkMA, USA; ^3^Organizational Behavior, Case Western Reserve UniversityCleveland, OH, USA

**Keywords:** vision, mood, engagement, emotional intelligence, individual differences, psychological climate

## Abstract

As organizational leaders worry about the appalling low percentage of people who feel engaged in their work, academics are trying to understand what causes an increase in engagement. We collected survey data from 231 team members from two organizations. We examined the impact of team members’ emotional intelligence (EI) and their perception of shared personal vision, shared positive mood, and perceived organizational support (POS) on the members’ degree of organizational engagement. We found shared vision, shared mood, and POS have a direct, positive association with engagement. In addition, shared vision and POS interact with EI to positively influence engagement. Besides highlighting the importance of shared personal vision, positive mood, and POS, our study contributes to the emergent understanding of EI by revealing EI’s amplifying effect on shared vision and POS in relation to engagement. We conclude by discussing the research and practical implications of this study.

## INTRODUCTION

Employee engagement has quickly become an important construct in organizational studies (e.g., Crawford et al., 2010; Rich et al., 2010; Gruman and Saks, 2011; Saks and Gruman, 2014). Empirical research suggests that employee engagement drives a number of positive individual and organizational outcomes (Saks and Gruman, 2014), including, for example, job performance (Rich et al., 2010), job satisfaction (Saks, 2006), and helping organizations reach their potential through business growth and profitability (Saks, 2006; Macey et al., 2009). Moreover, employee engagement is viewed as a source of competitive advantage (Kular et al., 2008), has become a catalyst for rethinking performance management systems (Gruman and Saks, 2011), and is used as a tool for improving talent management (Macey et al., 2009).

In spite of what we have learned so far about employee engagement, there is still a clarion call for more work to be done (e.g., Christian et al., 2011; Saks and Gruman, 2014). One under investigated area relates to the possible antecedents of employee engagement. For example, Saks (2006) pointed out “…there is little empirical research on the factors that predict employee engagement” (p. 604). More recently, Macey and Schneider (2008) lamented that “potential antecedents and consequences of engagement…have not been rigorously conceptualized, much less studied” (p. 304).

Our purpose is to address the need for further research on the antecedents of engagement. We first define employee engagement and review the current research on its antecedents. In doing so, we show that little has been done to explore the complex socio-psychological antecedents of engagement. Next, we test the association of two psychological climate factors and organizational support with employee engagement and whether individual characteristics moderate the relationship with engagement. Specifically, we investigate the association of (1) shared personal vision and shared positive mood (climate factors), (2) perceived organizational support (POS), and (3) peer-rated employee emotional intelligence (EI) with organizational engagement. Finally, we discuss the research and practical implications and contributions of these results and propose directions for further research.

## THEORY AND HYPOTHESES

### ENGAGEMENT

To date the definition of engagement still lacks universal agreement (Kular et al., 2008), but most refer to Kahn’s (1990) definition, which denotes employee engagement as “the harnessing of organization members’ selves to their work roles” (p. 694). More recent definitions tend to define employee engagement as an emotional and intellectual commitment to the organization (see Saks, 2006) and a representation of the level of personal commitment employees are willing to make or to invest in their job (Macey and Schneider, 2008). Others have noted that employee engagement represents the amount of discretionary effort employees will exhibit in their job (Frank et al., 2004). Similar constructs to employee engagement have also been presented like “work engagement” (“a relatively enduring state of mind referring to the simultaneous investment of personal energies in the experience or performance of work,” Christian et al., 2011, p. 95) and “job engagement” (“the investment of an individual’s complete self into a role,” Rich et al., 2010, p. 617), resulting in some initial discussion exploring the difference between work, job and, employee engagement (see Christian et al., 2011).

Drawing on the denotation of engagement being role related, Saks (2006) suggested that there are work roles (job engagement) and the role of being an organizational member (organizational engagement) that comprise employee engagement. These two forms of engagement were operationalized by using items that assess an employee’s “psychological presence in their job and organization” (Saks, 2006, p. 608). For the present study, we explore how individual characteristics impact one’s commitment to and connection with their role as an organizational member. Our particular interest is the connection one feels with something relatively more distant from themselves (i.e., the organization) as opposed to something more in their direct control (i.e., the job). At a time when employee engagement is still in decline in the United States in spite of a recovering economy and when globally 40% of employees still report they are unengaged (Aon Hewitt, 2013), organizations are clamoring to figure out how to strengthen the connection between the organization and their employees. We are curious as to what individual characteristics drive a person’s commitment to and affiliation with the organization they join. Thus, we focus on organizational engagement (herewith called engagement or employee engagement) in this study.

#### Antecedents of engagement

Recently scholars have started to explore the potential antecedents to employee engagement. Drawing upon social exchange theory (Cropanzano and Mitchell, 2005), prior research has found that job-related factors such as job characteristics and organizational support positively influence engagement (Kahn, 1990; Saks, 2006; Kular et al., 2008). In one of the earliest studies to look at the antecedents of engagement, Saks (2006) suggested that POS, perceived supervisor support, reward and recognition, procedural justice, and distributed justice were possible antecedents. As noted by Saks and Gruman (2014), most of the work on the antecedents of engagement has focused on measuring perceived work conditions, “neatly organized as job demands and job resources” (p. 167). These job demand (e.g., job task) and job resources (e.g., job control, job autonomy, job feedback, etc.) variables are not without their limitations (Saks and Gruman, 2014). For example, much of the research on employee engagement has focused on the job task, but “although the task is central, it is the degree to which the person can implement his or her preferred self in the work that is key” (Macey and Schneider, 2008, p. 21).

In contrast, Rich et al. (2010) found that value congruence, POS, and core-self evaluations had direct effects on engagement and that engagement mediated the impact of these three factors on the performance of firefighters. These scholars argue that engagement “reflects simultaneous investment of cognitive, emotional, and physical energies in such a way that one is actively and completely involved in the full performance of a role” (p. 622). We are interested in further understanding what “emotional energies” and individual characteristics, related to one’s “preferred self” and vision of a preferred, ideal future, lead to engagement. It is on this line of inquiry that our work builds.

Thus, while the aforementioned work on the antecedents of employee engagement represents a significant initial step toward understanding the drivers of engagement, it provides an incomplete explanation for the complex socio-psychological phenomenon engagement represents. If employee engagement is driven by an employee’s level of psychological presence in and emotional commitment to their role as organizational member, then a better understanding of the psychological drivers (e.g., aspirations, hopes, mood, etc.) and emotional and social capabilities of an employee should help identify other key antecedents of engagement. For example, it would seem that an exploration of antecedents that measure “the degree to which the person can implement his or her preferred self” (Macey and Schneider, 2008. p. 21) would be a critical antecedent of employee engagement.

### SOCIO-PSYCHOLOGICAL ANTECEDENTS

Psychological climate has been defined as the “perceptions that assess the significance and meaning of work environments to individuals” (James et al., 2008). A key influence on the perceptions employees have about the organization is the emotions employees feel (James et al., 2008). Emotions play a central role in nearly all action. Emotions excite interest, focus attention, alert the need for change, and move people to act (Fredrickson, 2001). Emotions also influence how people cope with challenge and threat, set new goals, learn new behaviors and draw on others for help or support (Fredrickson, 2001; Fredrickson and Branigan, 2005). The fields of positive psychology (Seligman and Csikszentmihalyi, 2000) and positive organizational scholarship (Cameron and Spreitzer, 2012) have accentuated a relational and contextual perspective to emotion: the extent to which an organization’s climate is emotionally positive or negative to an individual.

Intentional Change Theory (Boyatzis, 2008) postulates that both positive emotions play an important part of the push-pull affecting a person’s behavior through the neuro-endocrine, emotional, cognitive, and perceptual systems. In groups and organizations, the overabundance of positive to negative emotion forms a critical ratio as to the engaging nature of the environment, helping employees to open their minds and hearts, as well as increase the constructive aspects of social contagion (Fredrickson, 2001; Cameron, 2008). Further, others have suggested that the presence of positive personal dimensions of hope, vision, compassion, and overall positive mood are the essential components of an overall positive emotional climate (Fredrickson, 2001; Boyatzis, 2008; Cameron, 2008). These personal dimensions predict how open people are to others and others’ ideas, the degree to which they feel connected to and involved in both their work and with others, and how resilient they will be in moments of setback or failure. Thus, assessing these positive dimensions may present an important link to levels of employee engagement. Namely, as employees feel like the organization shares their personal vision for their work and feel positive about and supported by the organization for whom they work, they will likely be more engaged in their role as organizational members.

As a way of classifying these personal factors, Boyatzis (2008) grouped personal hopes, dreams, possibilities, positive outlook, and self-directed learning goals that make up one’s ideal self into what he called the “positive emotional attractor” (PEA, see Boyatzis, 2008). For the purposes of this study, we operationalize the PEA as *shared personal vision* and *shared positive mood*. We propose these two psychological climate factors will have a positive association with engagement. Shared vision captures the positive emotions employees feel about the organization’s view of the future and management’s commitment to reach a particular, clearly defined vision or purpose. Shared positive mood captures how employees feel about their work in the organization and the organization itself. High quality, positive relationships at work engender positive emotions, which can increase both individual and organizational commitment and effectiveness (Dutton and Ragins, 2007). Thus, we propose that these two climate factors enable shared, high quality connections with those one works with and to one’s work, to in turn promote higher engagement. In support of this direction of inquiry, one recent study found a strong relationship between employees’ shared positive mood and their level of engagement (Wijhe et al., 2011). We hypothesize the following:

#### Hypothesis 1

Shared personal vision positively associates with organizational engagement.

#### Hypothesis 2

Shared positive mood positively associates with organizational engagement.

### PERCEIVED ORGANIZATIONAL SUPPORT

Perceived organizational support is defined as “a general belief that one’s organization values [employees’] contributions and cares about their wellbeing” (Saks, 2006, p. 605; *cf* Rhoades and Eisenberger, 2002). In addition to the socio-psychological climate factors as possible key antecedents to engagement, Saks (2006) was the first to test the association between POS and engagement. He found support for a positive relationship between POS and engagement. Surprisingly, this line of inquiry has not been extended. We could not find additional studies that used POS as an independent variable and testing its relationship with engagement as a dependent variable.

Following our interests and the literature reviewed earlier, we retest Saks’ (2006) original hypothesis that proposed POS will have a positive association with engagement. More importantly, in addition to retesting Saks’ (2006) initial finding, we seek to extend his work in the present study by examining the relationship between EI, POS, and engagement (discussed in more detail later). In sum, we hypothesize the following:

#### Hypothesis 3

Perceived organizational support positively associates with organizational engagement.

### EMOTIONAL INTELLIGENCE

As a distinguishing individual capability, EI has caught the attention of scholars and practitioners alike (e.g., Goleman et al., 2002; Matthews et al., 2002; Ashkanasy and Daus, 2005; Mayer et al., 2008; O’Boyle et al., 2011; Walter et al., 2011). Some have acknowledged that much of the increased scholarly interest in EI is likely related to the mounting research showing the predictive and construct validity of EI (e.g., Ashkanasy and Daus, 2005; O’Boyle et al., 2011).

To date, most research has intelligence and motivation as relatively separated constructs (e.g., Kanfer and Heggestad, 1997; Kanfer and Ackerman, 2000; Schmitt et al., 2003). Certainly EI is not *g*, but EI combines affective and cognitive abilities, therefore cognitive processes are a significant part of EI. From their study on determinants of work motivation, Kanfer and Ackerman (2000) concluded: “The results of this study add to the growing body of evidence demonstrating the independence of individual differences in motivation and individual differences in intellectual abilities—as indexed by measures that aim primarily at assessing *g*” (p. 480). In their review, Schmitt et al. (2003) concluded that personality (not intelligence) was the primary predictor of motivation.

Under the current conceptualization of EI, there are three primary domains of research (Caruso, 2003). The first of these three treats EI as a set of interrelated intellectual abilities related to using emotional information. This domain is similar to models of general intelligence (Mayer and Salovey, 1997). The second domain is a trait approach that treats EI as a set of traits for adapting and coping. This domain is similar to models of personality and dispositional traits (Bar-On, 2000).

Finally, the third domain is a behavioral approach based on behavioral competencies. Similar to leadership competency models, this approach is related to combining affective and cognitive abilities. Under this third, behavioral domain (Boyatzis, 2009), EI is defined as the ability to be aware of self and use that awareness to influence one’s behavior. The resulting behaviors derived from strong EI are observable and measurable; therefore, this behavioral approach to EI has been operationalized using competencies that predict individual and team performance (see Offermann et al., 2004; Hopkins and Bilimoria, 2008; Boyatzis, 2009).

Competencies have been defined as learned capabilities that contribute to effective performance at work (McClelland, 1973; Boyatzis, 1982). A competency is any measurable characteristic of a person that differentiates level of performance in a given job, role, organization, or culture (Boyatzis, 1982). This competency approach to EI combines affective and cognitive abilities, but EI competencies are fundamentally different from competencies like technical skills, which rely solely on cognitive abilities based in the neocortex. Emotional intelligence is the ability to recognize, understand and use emotional information about oneself that leads to or causes effective or superior individual performance. Emotional intelligence exists when employees consistently demonstrate behaviors related to EI competencies, such as emotional self-awareness, emotional self-control, and adaptability, by drawing upon emotional information to influence behavior.

As an important component of employees’ emotional energy and preferred self, few studies have closely looked at association between interpersonal capability and employee engagement. Yet, some have suggested that behavioral competencies like communication skills and the ability to give upward feedback impact employee engagement levels (Kular et al., 2008). To our knowledge no one has looked at the emotional and social behaviors that might impact employee engagement; but, there is compelling evidence showing clear connections between EI and job performance (e.g., O’Boyle et al., 2011). Therefore, it makes sense that there may be a relationship between employee engagement and the emotional capability on the part of the employee. High EI should enable an employee to form, develop, and manage positive relationships with others (Goleman et al., 2002). Strong relationships at work should then lead to stronger connections with one’s organization (Dutton and Ragins, 2007).

#### Emotional intelligence as a moderator

There has been little research relating EI to psychological climate. Some initial results show that managers’ EI positively correlates with climate (Momeni, 2009). Further, “evidence does suggest that EI has potential to help scholars better understand leadership emergence, specific leadership behaviors, and leader effectiveness” (Walter et al., 2011, p. 55). As noted earlier, a positive climate can create an environment where people feel engaged and committed to their work and their organization. On the other hand, when the climate is negative and emotions are toxic, employees disengage from work, morale suffers, and performance drops (Frost, 2003). Still, as a meaningful, multi-faceted construct, engagement has not been sufficiently explored as it relates to EI.

How one performs in his or her job has been linked to the person’s level of engagement (e.g., Salanova et al., 2005; Ho et al., 2011) and employee engagement has been shown as a key predictor of individual, team, and business performance (e.g., Harter et al., 2002; Crawford et al., 2010; Gruman and Saks, 2011). Similarly, EI predicts job performance (O’Boyle et al., 2011). Latham and Pinder (2005) observed: “Research now shows that traits predict and/or influence job search and choice of job, as well as job performance and satisfaction. These traits include extroversion, conscientiousness, self-regulatory and self-monitoring strategies, tenacity, core self-evaluations, and goal orientation” (p. 488).

The behaviors that compose EI competencies help employees gain self-knowledge and engage in self-regulation to effectively facilitate relationships with others. Because EI is centered on understanding and managing self and employee engagement is about connecting oneself to one’s role as an organizational member, we surmise that EI will help facilitate the connection of self to an organizational role. We found one study (Ravichandran et al., 2011) that looked at the relationship between EI and work engagement and found no direct relationship.

We surmise that EI will impact engagement but, as noted earlier, prior research has not found this relationship to be one of a direct association. In contrast, we believe EI will have an “amplifying” role in its association with psychological climate factors, POS, and employee engagement. By “amplifying,” we mean to suggest EI increases the positive association of POS and psychological climate factors on engagement. We theorize that as an individual characteristic, EI does not have a direct association with organizational engagement because EI is centered on the self, particularly the self-awareness and self-management aspects of the self. On the other hand, POS, shared personal vision, and shared positive mood are constructs that assess how employees feel about the organization and their role as organizational members. This level of assessment of comparing self to one’s organizational role will be enhanced the more self-knowledge an employee possesses. As employees are clear about who they are, what they value, what they aspire to be, what they are good at doing, what type of support they want and need, for example, they can make more accurate judgments as to whether their goals and aspirations are being met.

Therefore, we believe EI will amplify the association between POS, mood, and vision and engagement. For example, EI can help an employee understand his or her personal vision and to assess the degree to which this vision is shared. EI likely empowers self-management to reconcile concerns about possible disconnects between an employee’s personal vision and the employee’s role as an organizational member. As the association between vision and one’s organizational role weakens, for example, EI can enable an employee to recognize and appreciate this disconnect and use self-management behaviors like emotional self-control and/or adaptability to rectify and strengthen the relationship. As EI increases, clarity and management of one’s vision and mood increases, which can in turn increase engagement. In sum, we propose that EI serves as a “check and balance” to amplify the association between POS and climate factors and engagement that would not be possible without the self-awareness and self-management capability that EI provides.

Therefore, we predict there will be positive association between EI, psychological climate, POS, and employee engagement such that EI will amplify the positive association shared vision, positive mood, and POS have on organizational engagement. In sum, the hypotheses that follow are designed to test the moderating role we believe individual characteristics (EI) play in amplifying psychological climate factors and POS that associate with engagement.

#### Hypothesis 4

Emotional intelligence positively increases the association of personal shared vision on organizational engagement.

#### Hypothesis 5

Emotional intelligence positively increases the association of shared positive mood on organizational engagement.

#### Hypothesis 6

Emotional intelligence positively increases the association of POS on organizational engagement.

## RESEARCH METHOD

### SAMPLE

Data were collected from one for-profit public company and one not-for-profit educational institution, both headquartered in a Midwestern state of the United States. These two consenting organizations agreed to provide full access to directly contact organizational members for possible participation in a web based data collection effort. In total, 638 engagement surveys were sent between the two organizations with a 44.7% response rate. Thus, 285 employees completed the engagement survey. The Institutional Review Board approved the Informed Consent and ethical conduct of the study at the third author’s university, and all protocols governing the use of human subjects were followed.

The for-profit company provided email addresses to all personnel in their Information Technology department while the not-for-profit institution provided email addresses for all of its administrative personnel. The web-based survey was administered over a 1 month period. The engagement, POS, and climate surveys started with the request for each employee to provide up to seven names of their co-workers that could rate the employee’s EI. An EI survey was then sent to each of the persons nominated. 798 co-workers completed the EI survey rating 238 study participants. Follow-up reminders were sent twice during the survey period.

After linking the climate, engagement, POS, and EI surveys, and retaining those cases that had complete data on all analysis variables, we obtained an analytic sample of 231 cases. Job tenure (time in *current job*) was measured ordinally on a scale ranging from 1 (“less than 1 year”) to 4 (“more than 10 years”). The modal response for job tenure was “between 1 and 5 years.” As noted in **Table 1**, employees in the for-profit organization had job tenure of 2.32, whereas those in the not-for-profit organization had job tenure of 2.73. Work experience was measured on a scale of 1 (“less than 1 year”) to 4 (“more than 10 years”). The modal response for each organization was “more than 10 years.”

**Table 1:** Demographic profile of the respondents by company type.

*Variable*	*Mean (SD)*
**Manufacturing (for profit)**	
Job tenure	2.32 (0.94)
Work experience	3.49 (0.83)
Salary	2.89 (0.79)
Gender (male)	0.65
*Role in Organization*	
Clerical^a^	0.04
Individual contributor ^a^	0.27
Management^a^	0.69
**Community college (not-for-profit)**	
Job tenure	2.73 (0.95)
Work experience	3.93 (0.25)
Salary	2.62 (0.78)
Gender (male)^a^	0.25
*Role in Organization*	
Clerical^a^	0.43
Individual contributor^a^	0.41
Management^a^	0.16

*Manufacturing *n* = 158; community college *n* = 73.*

*^a^For categorical variables the mean is proportion in category.*

**Table 1** shows that employees in the for-profit organization had a mean score of 3.46; whereas, those in the not-for -profit organization had a mean score of 3.93. Salary was measured on a scale of 1 “less than 20,000” to 4 “more than 100,000.” The modal response for each organization was “between 50,000 and 100,000.” In terms of salary, the for-profit organization had a mean score of 2.89; it was 2.62 for the not-for-profit organization. There was a substantial difference in the gender makeup of the two organizations; respondents from the for-profit organization were 65% male, whereas respondents from the not-for-profit organization were 25% male. Finally, we found that in the for-profit organization, 4% were clerical workers, 69% were individual contributors, and 27% were managers. For the not-for-profit organization we found that 43% were clerical workers, 16% were individual contributors, and 41% were management.

### MEASURES

Psychological climate, POS, and organizational engagement survey items used a five-point Likert scale, ranging from strongly disagree to strongly agree. We measured EI using a seventy-two item survey (discussed later). The climate factors were assessed with the PNEA Survey developed by Boyatzis based on earlier work (Boyatzis, 2008) and consisted of shared personal vision (eight items; e.g., “I feel inspired by our vision and mission” and “Management emphasizes a vision for the future”) and shared positive mood (five items; e.g., “This is a great place to work” and “Working here is a joy”). Alpha reliabilities for the two scales were as follows: *shared personal vision* (0.89) and *shared positive mood* (0.87).

The POS scale contained three items adapted from Saks’ (2006) scale. POS assesses the degree to which employees feel that the organization supports who they are [i.e., “My organization really cares about my well-being, “My organization strongly considers my goals and values,” and “My organization shows little concern for me” (reverse scored)]. The resulting POS scale had an alpha reliability of 0.88.

Using Saks (2006) engagement instrument, we retained four items to measure *organizational engagement* (e.g., “Being a member of this organization is exhilarating for me” and “Being a member of this organization is captivating”). The resulting scale had an alpha reliability of 0.90.

The EI variables were derived from the *emotional and social competence inventory* (*ESCI*), a 360-degree (or multi-rater) assessment (Boyatzis and Goleman, 2007). The test has shown desirable reliability and validity (Wolff, 2007), good model fit, and convergent and divergent validity at the scale level in a sample of more than 67,000 test takers (Boyatzis and Gaskin, 2010). A variety of performance and job outcome validation studies are reviewed for this test and its earlier versions in Boyatzis (2009).

The *ESCI* is designed for an individual employee’s manager(s), peers, and subordinates to rate the employee on 72 items. The survey items measure 12 distinct emotional and social competencies. As noted earlier, we invited study participants to select up to seven peers to rate them.

For the current study, we only used the EI scales. Because we were assessing aspects of the interpersonal climate through the perception of shared vision and POS, we believed we would likely have some overlap with the social intelligence behaviors and risk multicollinearity with the personal vision, POS and engagement measures. As a result, we chose to focus on the EI competencies rather than the SI competencies in our study. Further, when reviewing prior research, we concluded that among the EI competencies, emotional self-awareness seems to be predominantly an internal observation. This would make others’ observation of a team member’s emotional self-awareness more of a projection or attribution based on features other than observed behavior.

Conceptual logic based on past EI research (e.g., Taylor and Hood, 2011) indicates that these dimensions could be further combined into EI variables. We combined *adaptability/positive outlook*, *achievement orientation,* and *emotional self-control* to form a measure of EI. The resulting scale had an alpha reliability of 0.89.

To test the research hypotheses, we estimated path models using AMOS with simultaneous estimation of engagement. We tested the moderation hypotheses (i.e., H4–H6) using interaction terms.

Because there has been little attention devoted to the antecedents of engagement, there is not much theory to suggest which control variables may be most important. Given engagement is strongly connected to how one feels with their experience at work, it made sense for us to control for the type of organization one works for (for-profit versus not-for-profit), the type of work one does (e.g., clerical versus managerial), and the amount of time in one’s current role. We also chose to control for how much money an employee earns since one report noted that pay is a key driver of employee engagement (Aon Hewitt, 2013). Finally, we also empirically examined the relevance of gender and years of work experience as potentially important control variables, suggested by Cohen et al. (2003). In sum, all estimated models control for work experience, job tenure, salary, job type, organizational type, and gender.

Bivariate correlations, means and standard deviations for the analysis variables are presented in **Table 2**.

**Table 2:** Means, standard deviations, and correlations for the studied variables^a^.

	Mean	SD	1	2	3	4
(1) Shared personal vision	4.02	0.73				
(2) Shared positive mood	4.37	0.68	0.61**			
(3) Organization engagement	3.80	0.84	0.59**	0.65**		
(4) Perceived organizational support	3.99	0.97	0.61**	0.54**	0.59**	
(5) Emotional intelligence	4.20	0.48	0.23**	0.29**	0.20**	0.27**

^a^n = 231; **p < 0.01.

To further ensure the validity of the measures, we conducted a confirmatory factor analysis. The measurement model had 23 manifest variables specified as indicators of five latent constructs. All factor loading paths were positive and significant at the 0.001 level. Our measurement model for subsequent analysis had a good fit (Chi square 470, 219 df, IFI 0.930, CFI 0.929, RMSEA 0.071). The model had a PCFI of 0.737, indicating that the model was parsimonious and had acceptable fit.

## RESULTS

**Table 3** displays the results of our hypotheses testing. In all models, we added interaction terms separately to the model to aid interpretation and reduce concerns of multicollinearity.

**Table 3:** Unstandardized regression coefficients for organizational engagement.

	Model 1	Model 2	Model 3	Model 4
Company	-0.02 (0.11)	-0.02 (0.11)	-0.02 (0.11)	-0.03 (0.12)
Gender	0.01 (0.09)	0 (0.09)	0.01 (0.09)	0 (0.09)
Clerical	0.11 (0.14)	0.06 (0.14)	0.11 (0.14)	0.08 (0.14)
Manager	0.19 (0.10)	0.19 (0.10)	0.19 (0.10)	0.20 (0.10)*
Work experience	0.03 (0.07)	0.04 (0.07)	0.03 (0.07)	0.04 (0.07)
Time in current role	0.06 (0.05)	0.08 (0.04)	0.06 (0.05)	0.08 (0.05)
Salary level	-0.01 (0.07)	-0.02 (0.07)	-0.01 (0.07)	-0.02 (0.07)
Emotional intelligence	-0.06 (.09)	-1.29 (0.44)**	-0.09 (0.48)	-0.68 (0.29)*
Shared personal vision	0.24 (0.08)**	-1.08 (0.47)*	0.24 (0.08)**	0.25 (0.07)**
Shared positive mood	0.46 (0.08)**	0.46 (0.08)**	0.43 (0.46)	0.46 (0.08)**
Perceived organizational support	0.24 (0.05)**	0.26 (0.05)**	0.24 (0.05)**	-0.43 (0.31)
EI × shared personal vision		0.31 (0.11)**		
EI × shared positive mood			0.01 (0.11)	
EI × perceived organizational support				0.16 (0.07)*
Constant	-0.17 (0.44)	4.87 (1.83)**	-0.06 (1.96)	2.26 (1.19)
*R*^2^	0.54	0.56	0.54	0.55

n = 231. Standard errors are in parenthesis. *p < 0.05; **p < 0.01.

Model 1 in **Table 3** is the main effects model (i.e., no interaction terms added). The main effects model allowed us to determine which variables have direct effects on engagement. As reported earlier, prior research (Ravichandran et al., 2011), has not found EI to have a direct effect on engagement. We found a similar result in that model 1 shows EI (*b* = -0.06, *p* > 0.05) does not have a significant association with organizational engagement. On the other hand, hypotheses 1, 2, and 3 are supported in that shared personal vision (*b* = 0.24, *p* < 0.01), shared positive mood (*b* = 0.46, *p* < 0.01), and POS (*b* = 0.24, *p* < 0.01) all have positive, significant associations with engagement. Finally, Model 1 explains 54% of the variability in organizational engagement.

**FIGURE 1:**
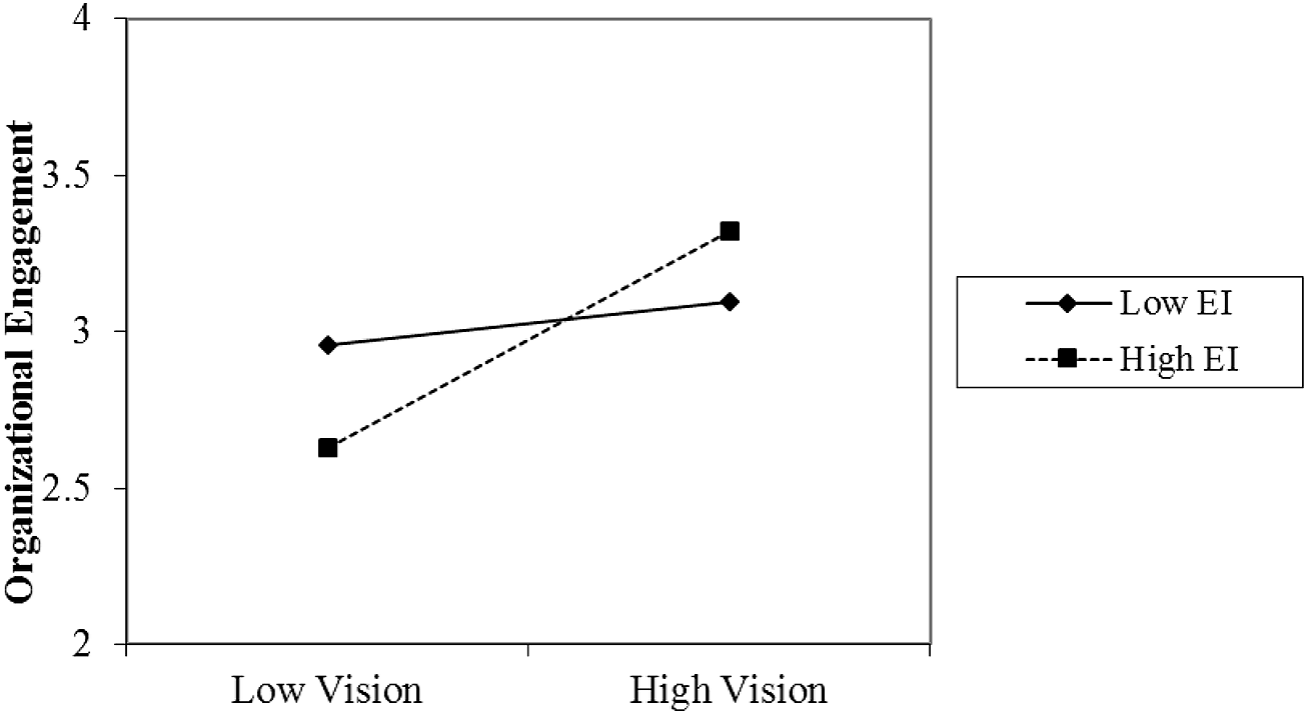
**The effect of shared personal vision on organizational engagement by high and low emotional intelligence (Model 2)**.

Model 2 adds the interaction terms of EI*shared personal vision to the model. **Table 3** shows that EI*shared personal vision has a significant positive association with organizational engagement (*b* = 0.31, *p* < 0.01). This coefficient suggests that increasing levels of EI amplify the relationship between shared vision and engagement; thus, we find support for hypothesis 4. **Figure 1** shows this interaction graphically. As can be seen from **Figure 1**, the slope for individuals with high levels of EI is steeper than the slope for individuals with lower levels of EI. In fact, **Figure 1** indicates that individuals with high EI and low shared vision are less engaged in their organizations; yet, individuals with high EI and high shared vision are more engaged.

Model 3 in **Table 3** adds the interaction terms of EI*shared positive mood to the model. Model 3 shows that EI*shared positive mood does not have a significant association with organizational engagement (*b* = 0.01, *p* > 0.05). As a result, we rejected hypothesis 5.

**FIGURE 2:**
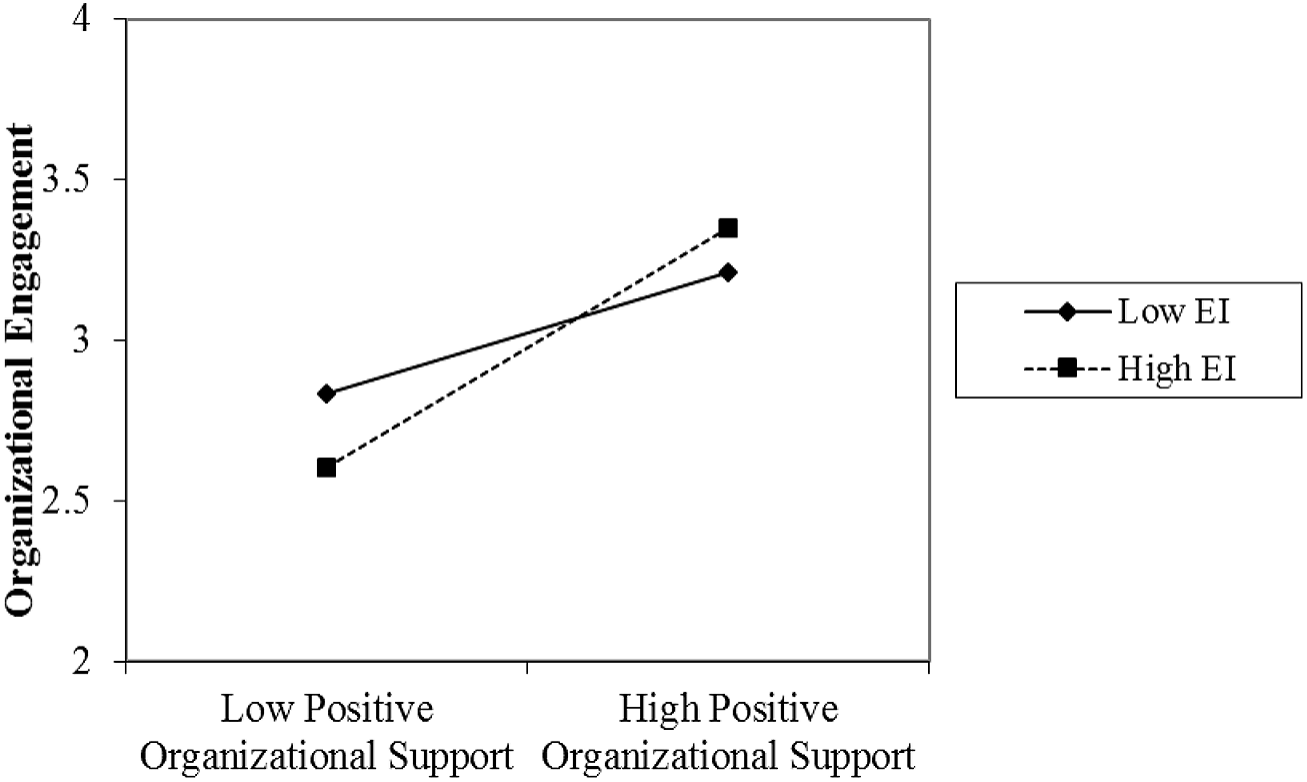
**The effect of perceived organizational support on organizational engagement by high and low emotional intelligence (Model 4)**.

Model 4 in **Table 3** adds the interaction terms of EI*POS to the model. EI*POS does have a significant positive association with organizational engagement (*b* = 0.16, *p* < 0.05). This coefficient suggests that increasing levels of EI amplify the relationship between POS and engagement. As a result, we accepted hypothesis 6. **Figure 2** shows this interaction graphically. As can be seen from **Figure 2**, the slope for individuals with high levels of EI is steeper than the slope for individuals with lower levels of EI. **Figure 2** indicates that individuals with high EI and low POS are less engaged in their organizations; yet, individuals with high EI and high POS are more engaged.

In sum, we found that peer-rated EI moderates the association of shared personal vision and POS with organizational engagement but does not do so with shared positive mood.

## DISCUSSION

### THEORETICAL CONTRIBUTIONS

Our primary contribution is we have confirmed POS’s impact on engagement and have introduced two additional antecedents (shared personal vision and shared positive mood) to engagement worthy of additional further research. We have extended prior theory by considering the association individual characteristics (EI), POS, and psychological climate factors (i.e., shared personal vision and shared positive mood) have with organizational engagement. Our research contributes to the understanding of engagement by revealing shared vision and shared positive mood have positive, direct associations on engagement. As an additional highlight of our results, this is the first study, to our knowledge, that presents EI as having an amplifying relationship between our predictor and outcome variables. Most prior studies on EI have only explored its role as an independent or dependent variable.

Our research emphasizes engagement’s role as a construct that is self-driven. This can be seen from Kahn’s (1990) original definition of employee engagement: “the harnessing of organization members’ selves to their work roles” (p. 694). Even the construct we drew upon for our measure denotes engagement in a similar way: the connection one feels in his or her role as an organizational member. This assesses the degree to which *individuals* are “attentive and absorbed” in their work (Saks, 2006). The self-awareness and self-management dimensions of EI work with psychological climate factors to activate an employee’s ability to harness the self to one’s organization, but it seems EI does not do that by itself. Although EI alone is about awareness and management of self, it appears insufficient to directly harness the self to one’s organization.

As we noted at the start of this paper, very limited work has been done to examine the relationship between EI and engagement. We found only one study that explored these relationships (Ravichandran et al., 2011). Although the researchers used a different measure of EI (i.e., Schutte et al., 1998) and a different measure for engagement (i.e., Schaufeli et al., 2006) than the measures used in this study, they too did not find a significant direct effect between EI and engagement. EI may drive job performance (O’Boyle et al., 2011), but, as we found, it does not seem to directly drive engagement. With only these two studies assessing the direct association of EI on engagement, we see the relationship between engagement and EI as an important area for further research.

We also proposed EI would interact with psychological climate factors such that EI would have an amplifying effect on the relationship between climate factors and engagement. In fact, our results indicate that for organizational engagement, shared vision indeed has an amplifying pattern whereby when individuals have high EI, shared vision strengthens the level of engagement. Those with high EI would, as our data show, be dissatisfied and therefore less engaged in a relational climate with low shared vision. In sum, EI is an important moderator in amplifying the association of shared vision with organizational engagement. As noted earlier, Boyatzis (2009) and others (Goleman et al., 2002) defined EI as the ability to understand self and to use that understanding to effectively manage self. It is conceivable that EI enables greater clarity and understanding of climate factors and assists potentially distant climate factors, like shared vision, to become internalized and valued such that the interaction with EI produces greater organizational engagement.

Perhaps individuals use their EI to clarify and make use of their shared personal vision to strengthen their commitment to and connection with their organization. Organizational engagement items such as “one of the most exciting things for me is getting involved with things happening in this organization,” connote a connection beyond the functional area of one’s job. Therefore, it is understandable to see a significant and positive relationship between organizational engagement and the interactions of EI with POS and shared vision. EI may help the self (with its values, goals, aspirations, hopes, etc.) clarify how the vision and purposes of the organization relate to the self and to then, in turn, increase the connection one feels to the organization.

For years organizations have created vision and mission statements and research has supported the importance of their use in organizations (e.g., Baum et al., 1998); indeed, research has shown that vision statements are related to organizational growth and performance (e.g., Baum et al., 1998; Kantabutra and Avery, 2010). One of the great leadership challenges is how to help employees connect with an organization’s vision such that the vision becomes shared and intrinsically accepted (Kouzes and Posner, 2007). Our findings suggest that a key to that internalization of the shared vision is the level of EI the employees possess. As organizations invest in the EI development of their employees they are also likely enabling those employees to further link their personal vision with the vision of the organization to in turn increase employee engagement. Similarly, through its self-knowledge building capability, EI helps employees realize what type of support they want and need from an organization. In doing so, EI amplifies the association between POS and engagement. Without high EI, employees may struggle to know themselves and manage themselves effectively (e.g., via decisions they make about their jobs and careers) enough to recognize what type of organizational support is most important to them. If you don’t know what you value and what goals are important to you, you are less likely to know if the organization you work for cares about your values and goals.

Finally, shared positive mood had the strongest direct association on organizational engagement, but it did not have an interactive effect with EI on engagement. Employees’ shared positive mood is a driver that harnesses the self to the employees’ work in positive ways. Thus, it is no surprise to find that when shared positive mood is high, employees feel more engaged. As noted earlier, Wijhe et al. (2011) also found a strong relationship between employees’ shared positive mood and their level of engagement. This is clearly an important area for additional research.

In terms of the insignificant association between EI and mood and engagement, prior research has argued that mood, in contrast to emotions, “are weaker or diffuse, last longer,…and tend to elicit a wider range of cognitive and behavioral responses than do emotions because they are not targeted toward specific causes” (Rhee and Yoon, 2012, p. 224n1). In contrast, emotions (1) work to provide us specific information about what our goals are and where we stand in achieving our goals and (2) provide “amplification of goal-directed motivation” (Batson et al., 1992, p. 308). EI is focused on being aware of and managing *specific* emotions. Our items for shared positive mood assessed general feeling states from individuals about where they work (e.g., “I enjoy working here,” “Working here is a joy,” etc.). As a relational construct, EI may do little to directly influence the diffuse opinions about work.

### LIMITATIONS

Even though the findings in our study generally support five out of six of the hypotheses, the study is not without limitations that should be considered in the design of future research. First, this study’s sampling procedure was not random, opening the possibility of selection bias, as we have no way of knowing if the responses to our survey are different than those that chose not to complete the survey. Selection bias reduces the external validity of this research. Further limiting the external validity of this research is the ability to generalize differences between for-profit and not-for-profit organizations due to our sampling strategy. That is, we did not take a sample of for-profit and not–for-profit organizations but chose two that were willing to participate. Differences found between these organizations could be due to specific characteristics of these two organizations rather than differences between the for-profit and not-for-profit sectors.

Second, due to the large number of questions being asked of each respondent, we were unable to ask a full set of demographic questions (e.g., educational attainment). This characteristic of the data collection could lead to omitted variable bias if an unobserved factor is related to our independent and dependent variables.

Third, upon examination of frequency tables, histograms, box plots and distributional statistics to determine the shape of the distributions of the individual items, we found that items comprising the engagement and climate scales were not normally distributed around their mean, rather most items in this survey showed distributions with negative skewness and high kurtosis values, a pattern caused by many respondents answering on the high end of each item. Given the non normal distributions, we will interpret inferential results (i.e., any significant tests) with caution, and it should be noted that the limited range and variance might under estimate true population associations among the variables in our model.

Finally, a common method bias analysis indicated that there was a possibility of common method bias. We suspect this is related to the non-normal variable distributions. Given this possibility, we again interpret results with caution and view it as a limitation of this research. Analyses of path models accounting for common method bias were estimated and showed no substantive differences from the results presented above.

### IMPLICATIONS FOR FUTURE RESEARCH

Our research findings serve as an invitation toward a new agenda in vision, POS, EI, and engagement research. As an individual characteristic, EI plays an important role in the relationship between engagement, POS, and shared vision. We see this as a vital area for further study. As others have done recently (e.g., Rich et al., 2010), our conceptualization of engagement is one of engagement as a source of motivation.

Future research should seek to understand how shared vision, mood, and POS build on the relational aspects of engagement. In this study we examined EI. Social intelligence relies on behaviors that help people understand others and manage others effectively. Engagement has relational qualities given an organization’s culture is a composite of the shared values and vision of many. It would be interesting to see if social intelligence also plays an amplifying role in its association with the variables used in this study.

We join the call for additional research on the antecedents of engagement, but our work has called attention more directly to the importance of socio-psychological factors that may drive engagement. More work is needed to understand EI as a moderator to the relationship between shared vision and POS and engagement. The importance and impact of positive emotions in organizational life is a growing area of organizational scholarship (Cameron and Spreitzer, 2012). Positive emotion inducing constructs like shared vision and shared mood and individual characteristics like EI should be further investigated given the role they play in engagement. For example, as we noted earlier, to date no one has studied the potential amplifying role EI may have with shared vision on engagement. Research examining these relationships with larger more diverse organizational samples would be a particularly important elaboration of the analyses presented here.

Leaders must be concerned with engagement in the workforce. Having a clear awareness of engagement levels is a useful predictor of behavior and performance. Future research should continue to examine engagement as an important aspect of organizations. The current study found interesting differences in the determinants of engagement (vision, mood, and POS versus EI) suggesting different processes might lead individuals to be engaged to their organizations. Future research should attempt to clarify why these processes are different and what this means for managers trying to lead their workforce in an optimum way.

### IMPLICATIONS FOR PRACTICE

The purpose of our study was primarily focused on testing empirical relationships between individual characteristics, organizational support, and psychological climate factors and engagement. On the other hand, our findings do lend themselves to several practical implications. First, one of the challenges in organizations today is how to help employees believe in and become loyal to the organizational vision, see their job as important, and trust that the organization supports its employees (Kouzes and Posner, 2007). Certainly the level of authenticity of management and whether their efforts to garner employee trust and commitment are at the expense of their employees or in support of them matters, but at a time when employee loyalty is reportedly slipping worldwide (Brotherton, 2012), our findings offer help. We find that EI assumes an amplifying role for shared vision and engagement. Therefore, organizations that work to hire employees with high EI and to foster EI development in their organizations will strengthen the ties between important employees’ vision, the degree they feel supported by the organization, and their level of engagement to the organization.

Next, our research also exposes the importance of fostering the emotional and socio-psychological factors of climate. Our findings reveal empirical evidence of their impact on engagement at a time when the relationship between engagement and performance are becoming well documented (e.g., Macey et al., 2009; Rich et al., 2010). Therefore, organizations should work to hold up these climate factors as important psychological elements as they do the more cognitive-based constructs like strategy, forecasting, planning, and budgeting, for example. In doing so, organizations will begin to leverage their employees engagement as an important competitive advantage.

## CONCLUSION

This study highlights the importance of shared personal vision, shared positive mood, and POS as key areas for further research on engagement. This study also contributes to our growing understanding of EI by displaying EI’s amplifying effect on shared vision and POS in relation to engagement. We now invite others to join the call for understanding these and other important antecedents to engagement.

## Conflict of Interest Statement

The authors declare that the research was conducted in the absence of any commercial or financial relationships that could be construed as a potential conflict of interest.
